# Reactive Lymphoid Hyperplasia of the Liver: A Clinicopathological Study of 7 Cases

**DOI:** 10.1155/2012/357694

**Published:** 2012-07-30

**Authors:** Lei Yuan, Youlei Zhang, Yi Wang, Wenming Cong, Mengchao Wu

**Affiliations:** Second Department of Hepatic Surgery, Eastern Hepatobiliary Surgery Hospital, Second Military Medical University, 225 Changhai Road, Shanghai 200438, China

## Abstract

*Background*. Reactive lymphoid hyperplasia (RLH) of the liver is a benign focal liver mass that may mimic a malignant liver tumor. Although rarely encountered in clinical practice, it often poses diagnostic and management dilemmas. *Methods*. Cases diagnosed as hepatic RLH between January 1996 and June 2011 were investigated in a retrospective study. Clinicopathological features as well as follow-up information of the cases were studied. *Results*. A total of seven cases of hepatic RLH were investigated, with a median age of 46 years (range: 33–76 years). Hepatic RLH was accompanied by concomitant diseases in some patients. The average size of hepatic lesions of our cases was 45 mm (range: 15–105 mm). All of the cases were not accurately diagnosed until confirmed by pathological findings, and surgical resections were performed for all. Postoperative course was uneventful for all of the patients during followup. *Conclusions*. RLH of the liver is a rare benign disease with a female predilection of unknown etiology. It is very difficult to correctly diagnose this disease without pathological results. Subtle differences on radiological findings of it may be helpful for differential diagnosis from other diseases. Curative resection of the lesion is suggested for the treatment of this disease.

## 1. Introduction

Reactive lymphoid hyperplasia (RLH), also termed as nodular lymphoid lesion [1, 2] or pseudolymphoma [3–8], is a rare benign condition which forms a liver mass typically infiltrated by massive heterogeneous mature lymphoid cells without prominent nuclear atypia, with formation of follicles and germinal centers. Up to now, there have been only scattered case reports about hepatic RLH [1–24]; the paucity of information about its clinicopathological features poses diagnostic and management dilemmas. Manifested as a focal liver mass, hepatic RLH may mimic a malignant liver tumor, often giving rise to misdiagnosis. Therefore, we reviewed our experience in a series of 7 patients with hepatic RLH who were treated by surgical resection. This was the largest series of cases of this disease so far, and a detailed evaluation of its clinical features, radiologic characteristics, and pathologic findings will assist in future diagnosis of this disease.

## 2. Methods

The data of all cases diagnosed as hepatic RLH or pseudolymphoma and treated by surgery in Eastern Hepatobiliary Surgery Hospital, a tertiary university hospital in China, between January 1996 and June 2011 were obtained from the computerized files. Results of imaging studies such as ultrasonography (US), computerized tomography (CT), and magnetic resonance imaging (MRI), the clinicopathological findings and the outcomes of followup of the patients were retrospectively reviewed. 

All the patients underwent surgery with complete resection of lesion(s). The resected specimens were fixed in 4% buffered formalin, processed, and embedded in paraffin. Histologic sections were stained with hematoxylin and eosin. Immunohistochemistry of paraffin sections was carried out using EnVision system (a two-step staining technique) as described previously [25]. Negative control slides omitting the primary antibodies were included in all assays. The evaluation of the immunohistochemical findings was performed without any knowledge of the clinicopathologic data by two independent clinical pathologists.

After surgery, all the patients were monitored by serumtumor markers, abdomen US, and chest X-ray every 1 to 3 months. If recurrence suspected, the patients underwent CT or MRI for further evaluation. The last followup was completed on December 31st, 2011 and the data were analyzed after the last followup.

## 3. Results

### 3.1. Patients' Demographics

From January 1996 to June 2011, a total of 45,000 patients with occupied lesion(s) in liver were admitted to our hospital for partial hepatectomy. In 7 of them, hepatic RLH was confirmed by pathological examination of resected specimens. There were 6 females and 1 male, with a median age of 46 years (range: 33–76 years). The patients were either asymptomatic or had mild nonspecific symptoms such as abdominal discomfort. Hepatitis B surface antigen was negative in 6 female patients, but positive in the male patient who was accompanied by cirrhosis. Hepatitis C virus antibody was negative in all the patients. Tumor markers including AFP, CEA, and CA19-9 were within normal range in all cases. Hepatic function tests and blood routine test were normal. Hemangiomas happened to occur simultaneously with RLH in two patients, and gall bladder calculi and fibroadenoma of breast are found in two female patients. In this series, the sizes of hepatic lesions varied from 13 × 15 mm to 105 × 65 mm on US scan, and the lesion of the male patient progressed during three years before operation (from 1.0 × 1.2 cm to 4.0 × 2.0 cm). Preoperatively, hepatocellular carcinoma (HCC) was suspected in 6 patients and intrahepatic cholangiocellular carcinoma in the other one.

### 3.2. Surgical Outcomes

All the patients underwent curative local resections, and the postoperative course was uneventful in all cases. After surgery, no patient received subsequent therapy for hepatic RLH. Recurrence did not occur in any of our patients during a median followup of 68 months (range: 6–228 months).

### 3.3. Features on Imaging Studies

**Figure 1:**
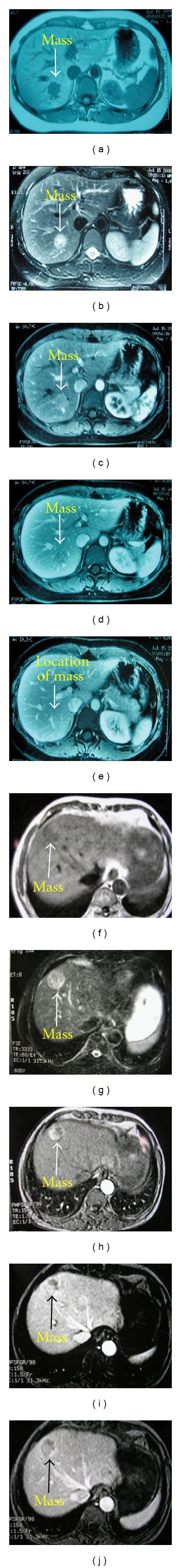
Comparison of appearances of hepatic RLH (a–e) with hepatocellular carcinoma (f–j) on fast spoiled gradient recalled echo (FSPGR) MRI. (a) On unenhanced T1-weighted image, the lesion is hypointense signal relative to normal liver parenchyma. (b) The lesion is hyperintense signal in the same location on T2-weighted image. (c) The lesion is enhanced in the arterial phase. (d) The lesion is hypodense in the portal phase. (e) The lesion is unclear in the delayed phase. (f) On plain T1-weighted imaging scan, the lesion is hypointense signal relative to normal liver parenchyma. (g) The lesion is hyperintense signal in the same location on T2-weighted image. (h) The lesion is significantly enhanced in the arterial phase. (i) The lesion is hypodense in the portal phase. (j) The lesion became more hypodense in the delayed phase.

All the lesions of RLH were revealed as well-defined hypoechoic masses on US examination. The lesions were presented as hypodense masses on plain CT scan, slightly enhanced in the arterial phase, hypodense in the portal phase and the delayed phase as compared with liver parenchyma. MRI depicted hepatic RLH as nodular lesions with hypointense signal masses on T1-weighted imaging (Figure 1(a)), hyperintense signal on T2-weighted imaging (Figure 1(b)), and the lesions were moderately enhanced in the arterial phase (Figure 1(c)) followed by hypodense areas in the portal phase (Figure 1(d)) and unclear in the delayed phase (Figure 1(e)).

### 3.4. Pathologic and Immunohistochemical Analysis

**Figure 2:**
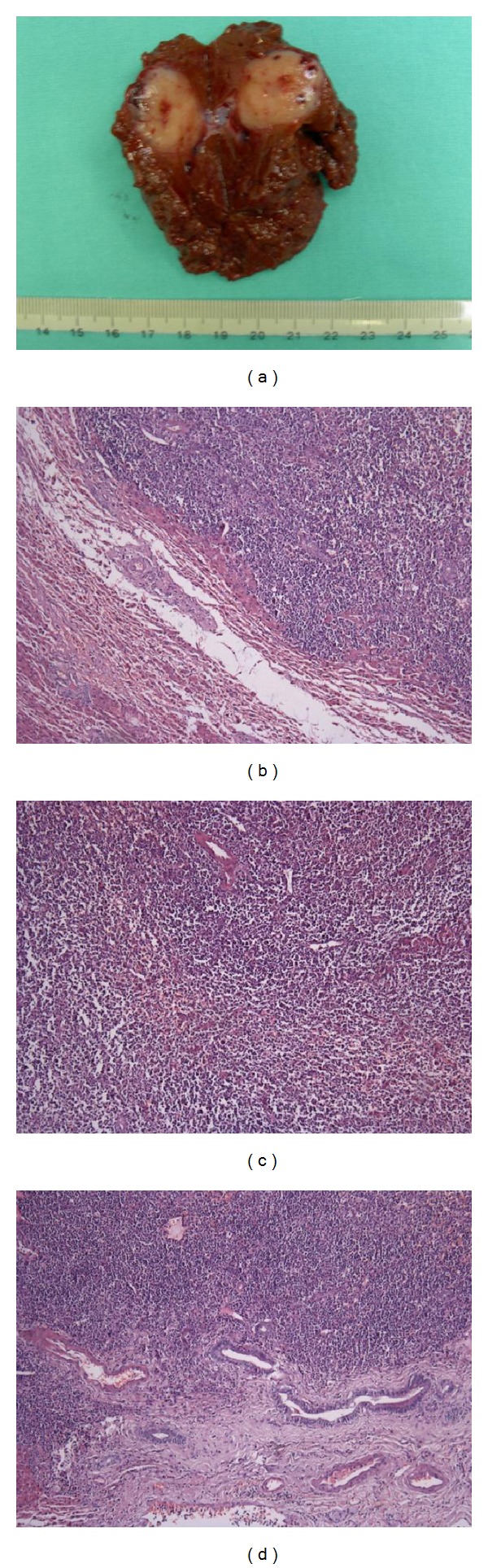
(a) Macroscopically, a cut section of the resected liver showed a well-circumscribed, encapsulated, yellow-white nodular lesion in segment 6, with small areas of hemorrhage and necrosis. (b) Microscopically, the lesion was well demarcated and encapsulated, and comprised a massive infiltration of mature lymphoid cells, forming follicles and germinal centers (H&E staining, 100x magnification). (c) The infiltrated lymphoid cells was mature and heterogeneous, with no nuclear atypia or polymorphism (H&E staining, 100x magnification). (d) Note the lymphocytic infiltration in the portal tracts around the lesion (H&E staining, 100x magnification).

**Figure 3:**
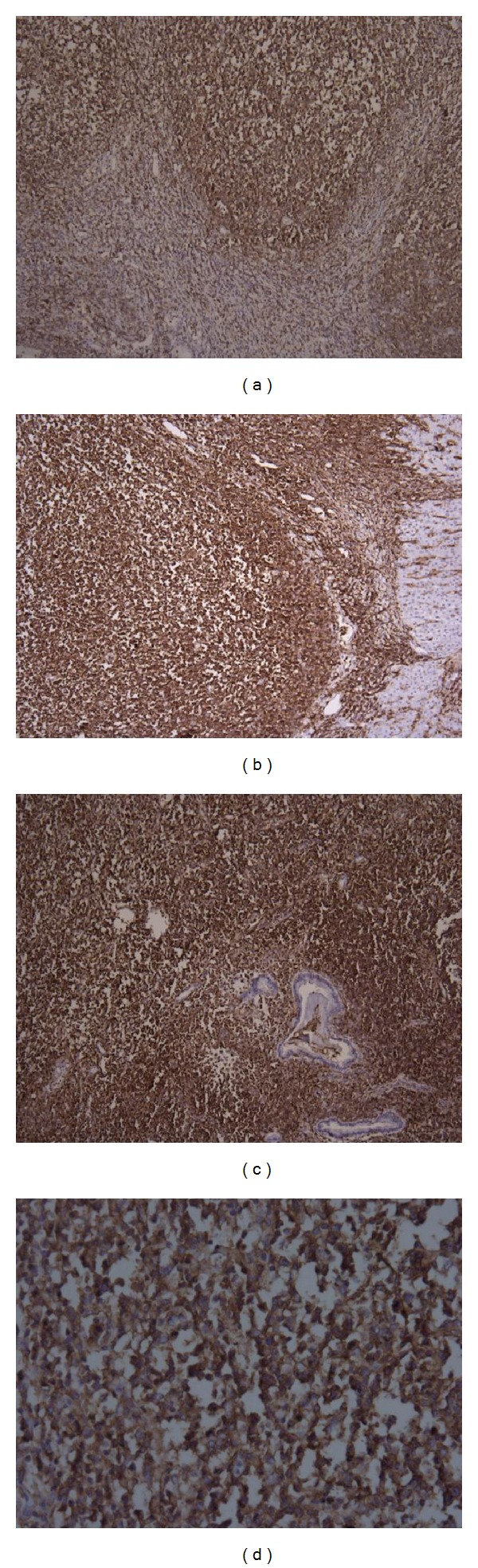
(a) Immuno.histochemistry showed that germinal centers mainly comprised CD20 (+) B lymphocytes (100x magnification) (b) Germinal centers mainly comprised LCA (+) lymphocytes (100x magnification) (c) T lymphocytes in interfollicular area and surrounding germinal centers were CD45RO (+) (100x magnification) (d) Germinal centers mainly comprised CD20 (+) B lymphocytes (400x magnification).

On the cut sections, the lesions were grossly distinct firm nodules well demarcated from the surrounding liver tissue, sometimes encapsulated (5/7), with small areas of hemorrhage and necrosis (3/7) (Figure 2(a)). Histologic findings were similar for all the patients. Seen as a well-delineated nodular area microscopically, the lesions comprised a massive infiltration of heterogeneous mature lymphoid cells with no prominent nuclear atypia (Figure 2(c)), forming follicles (varied in size and shape) and germinal centers (Figure 2(b)), and there was no evidence of a monoclonal B cell population. In addition, fibrous materials aggregating in parts of the lesions (4/7), lymphocytic infiltration in the portal tracts around the nodular lesion (5/7) (Figure 3(d)), and bile ductules (2/7) were seen within the lesion. Germinal centers mainly comprised CD20 (+) and LCA (+) lymphocytes (Figures 3(a) and 3(b)), while lymphocytes in the interfollicular area and surrounding germinal centers were CD3 (+) and/or CD45RO (+) (Figures 3(c) and 3(d)). A polyclonal pattern of IgH (immunoglobulin heavy chain) and TCR-*γ* (T-cell receptor gamma) gene rearrangements of lymphocytes in the lesion was found in most patients.

## 4. Discussion

**Figure 4:**
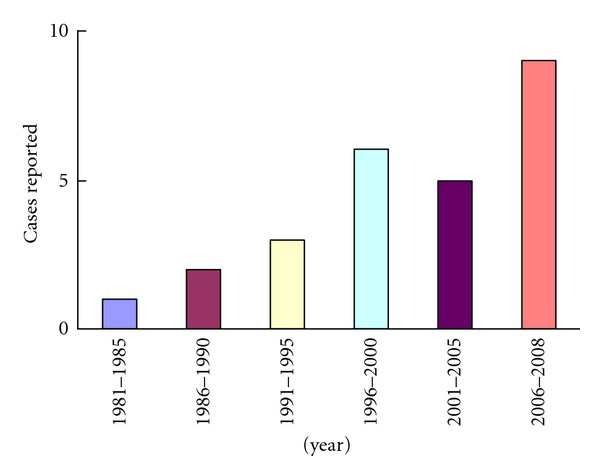
The incidence rate of this disorder seems to be increasing, calculated by the time when reported.

RLH is a benign condition that may occur, besides in liver, in the gastrointestinal tract [26], orbit [27], lung [28], skin [29], and thyroid [30]. Hepatic RLH was first reported by Snover et al. in 1981[8], and only 34 cases have been reported in the English literature until now [1–25, 31, 32]. We found 7 cases from 45,000 patients who underwent hepatectomy for liver masses, indicating the rarity of this disease. The incidence rate of hepatic RLH seems to be discovered increasing with time, as shown in Figure 4, perhaps for the advances in diagnostic imaging technologies and the accumulation of doctors' experience. However, the incidence rate might be underestimated due to doctors' limited understanding of this disease.

At present, this is the largest case series review of hepatic RLH. In total, 41 cases of hepatic RLH are reported, including 36 females and 5 males (median age of 57 years; range, 15–85 years). All hepatic RLH patients were adults except for one 15-year-old female. A female predilection of this disease was obvious in both the present series (6/7) and when considering the sum of all cases (36/41). 6 cases have multiple hepatic lesion of RLH, and there were 49 hepatic lesions altogether. The average size (in greatest dimension) of hepatic lesions of our cases was 45.0 mm (range: 15–105 mm), which was bigger than that (15.6 mm in average; range: 4–60 mm) of the reported cases. The background data and the clinical characteristics of these 41 patients were summarized in Table 1.

The exact etiology of this disease remains unclear. An association between the development of hepatic RLH and some disease such as autoimmune diseases [1, 4–7, 9, 11] and malignant tumors [3, 6, 10, 13, 15, 20, 22, 31] has been suggested to be most likely involved in previous reviews. However, we believe this is not convincing because such concomitant diseases were not found in this series, and the reviews are independent, intermittent case reports. 

It is very difficult to make a correct diagnosis of hepatic RLH before operation, as almost all of the cases including the reported have been misdiagnosed. Hepatic RLH has most frequently been misdiagnosed as HCC, as preoperative diagnosis are HCC (6/7) for our cases, while HCC (15/41) for all cases including the reported. Thus, differential diagnosis between hepatic RLH and HCC is important but difficult, given their similarity on radiological appearances. However, in our cases, we found subtle differences in radiological findings between hepatic RLH and HCC. On MRI scan, hepatic RLH is always moderately enhanced in the arterial phase (Figure 1(c)), hypodense in the portal phase (Figure 1(d)) and unclear in the delayed phase (Figure 1(e)); while HCC is remarkably enhanced in the arterial phase (Figure 1(h)), prominently hypodense in the portal phase (Figure 1(i)) and more hypodense in the delayed phase (Figure 1(j)). The most characteristic radiological findng is that hepatic RLH becomes unclear in the delayed phase after injection of contrast agents. In addition, HCC is always accompanied by hepatitis virus infection and elevated level of AFP in China, which can be added to differential clues. From all the cases, we conclude the following radiological clues to help to diagnose hepatic RLH: (a) well-defined hypoechoic mass on the US images [4–7, 9–12, 14, 17–19, 23, 24, 32–34]; (b) low-density lesion in plain CT phase, mild enhancement in the arterial phase, hypodense areas in the portal phase [1–7, 9, 10, 12–18, 23, 24]; (c) hypointense signal on MRI T1-weighted imaging, hyperintense signal on T2-weighted imaging, moderately enhanced in the arterial phase, hypodense areas in the portal phase and unclear in the delayed phase [2, 3, 6, 10–15, 17, 18, 24]; (d) hypervascularity on angiography [11]. Preoperative imaging findings of the lesions of all the cases are listed in Table 2.

It is difficult to definitely recognize hepatic RLH by routine histologic evaluation alone. Common morphologic features include a well-demarcated region, hyperplastic lymphoid follicles with active germinal centers, hyalinized trabecular structures, and lymphocytic infiltration in the portal tracts around the nodular lesion [1, 5, 6, 9–15, 31, 35]. However, all of these appearances are not enough to distinguish hepatic RLH from inflammatory myofibroblastic tumor and MALT lymphoma, given the similarity of morphologic features among them. Immunohistochemical and molecular genetic investigations are necessary to differentiate them [35]. In most hepatic RLH cases, germinal centers consist of polyclonal mature lymphocytes without cytologic atypia (mainly CD20 (+) and LCA (+)), and lymphocytes in the interfollicular area were predominantly CD3 (+) and/or CD45RO (+) (Table 3). In addition, polyclonal patterns of IgH and TCR-*γ* gene rearrangements of lymphocytes in the lesions were often observed (Table 3). However, in cases of primary hepatic MALT-type NHL [36–43], small B cells proliferating in lymphoid follicles displayed monoclonality. Although preoperative needle biopsy was applied in 5 reported cases [15, 17, 20, 35], it is not recommended because it cannot provide comprehensive details of results of immunohistochemical and molecular genetic studies. Also it is difficult to differentiate hepatic RLH from primary hepatic lymphoma only by needle biopsy [35].

Although hepatic RLH is generally thought to be benign, it might transform into malignant lymphoma [32], as reported in the lung [44] and stomach [45]. These early reports, however, were lack of the use of immunofluorescent and molecular techniques, and it is likely that these cases were in fact the early stage of primary lymphoma misdiagnosed as benign [46]. But evidence of progression from histologically benign, immunohistochemically polyclonal lymphoid infiltrates to malignant lymphoma in cutaneous pseudolymphoma has been reported in the literature [47]. Also we speculate hepatic RLH may grow with time, because the lesion of the male patient progressed during the three years before operation, similar to two reported cases [11, 18]. But different voices existed that hepatic RLH might have spontaneous regression, as in three cases [20, 35], diameter of the lesion had decreased during the followup without operation. However, it is not convincible because hepatic RLH was diagnosed by core needle biopsy in the three cases. Surgical resection is suggested both for treatment and for a definitive diagnosis of hepatic RLH even when pseudolymphoma is diagnosed preoperatively. Treatments for all the cases were surgical resection (7/7) in our series; while surgical resection (33/41), liver transplantation (2/41), percutaneous ethanol injection (1/41), core needle biopsy and observation (3/41) [20, 35], and unknown (2/41) in all cases including the reported. No complication related to surgical treatment happened. The prognosis of hepatic RLH is good, and all the patients treated by resection have shown no recurrence or progression to lymphoma.

In conclusion, hepatic RLH is a rare disease which mostly occurs in females, and the exact etiology of this disease is still unknown. Hepatic RLH has similar features with malignant liver tumors on radiological findings, but subtle differences such as “the lesion becomes unclear in the delayed phase on MRI scan with injection of contrast agents” can be found to be helpful for differential diagnosis. An accurate postoperative diagnosis of this disease depends not only on morphologic findings, but also on immunohistochemical analysis and molecular investigations. Given that hepatic RLH may grow with time or might even transform into malignant lymphoma, surgical resection is suggested, both for the safety and for good prognosis of the disease.

## Figures and Tables

**Table 1:** Background data and clinical characteristics of all cases including the reported.

Background data and clinical characteristics	
Variables	Cases
Age (Y)	
15–30	1
31–60	21
60–85	19
Sex	
Female	36
Male	5
Concomitant disease	
Chronic hepatitis	7
Sjögren's syndrome	1
CREST syndrome	1
Autoimmune thyroiditis	5
Malignant tumor	12
Hepatic hemangioma/FNH	3
PBC	4
DM	2
Immunodeficiency	2
Size in the greatest dimension of lesions (cm)	
*⩽*4	37
>4	4
Number of lesions	
Solitary	35
Multiple	6
Location	
Rt. lobe	21
Lt. lobe	13
Lt. lobe and Rt. lobe	2
NA	5
Treatment	
Surgical resection	34
Transplantation	2
CNB and PEI	1
CNB and observation	3
Autopsy	1

Rt: right; Lt: left; Seg: segment*；* NA: not available; PBC: primary biliary cirrhosis; FNH: focal nodular hyperplasia; DM: diabetes mellitus; PEI: percutaneous ethanol injections; CNB: core needle biopsy; NA: not available.

**Table 2:** Preoperative imaging findings of hepatic RLH of all cases including the reported.

Preoperative imaging findings	Cases
US	
Hypoechoic mass	7
CT	
Plain: hypodense	18
Arterial: significantly/slightly/peripherally enhanced	12
Parenchymal and portal: clearly/vaguely low	12
MRI	
Plain T1: low; T2: high	13
Arterial: highly/slightly enhanced	8
Portal and delayed: peripherally ring enhanced or clearly/vaguely hypointense	8
Angiography	
Hypervascularity	10

US: ultrasonographic; CT: computerized tomography; MRI: magnetic resonance imaging.

**Table 3:** Pathological characteristics of hepatic RLH of all cases including the reported.

Histological, immunohistochemical, and molecular findings	Cases
LIPTANL	22
Germinal center CD20/L26 (+)	26
Germinal center LCA (+)	6
Interfollicular area and surrounding germinal centers CD45RO/UCHL1 (+)	15
Area surrounding germinal centers CD3 (+)	11
Ductal structures at the periphery of the nodule CK7 (+)	5
Polyclonal in *κ* and *λ* light chain staining	26
Massive infiltration of heterogeneous mature lymphoid cells with no nuclear atypia, forming follicles and germinal centers	41

LIPTANL: lymphocytic infiltration in the portal tracts around the nodular lesion; NA: not available; L26 = CD20; UCHL1 = CD45RO.
